# Clinical-epidemiological characteristics and maternal-foetal outcomes in pregnant women hospitalised with COVID-19 in Venezuela: a retrospective study

**DOI:** 10.1186/s12884-022-05253-2

**Published:** 2022-12-05

**Authors:** Fhabián S. Carrión-Nessi, Mercedes P. Castro, Diana C. Freitas-De Nobrega, Augusto Moncada-Ortega, Óscar D. Omaña-Ávila, Daniela L. Mendoza-Millán, María V. Marcano-Rojas, Nayren J. Trejo, Isabella V. Virriel, Melynar Chavero, Natasha A. Camejo-Ávila, Alfonso J. Rodriguez-Morales, David A. Forero-Peña

**Affiliations:** 1 Biomedical Research and Therapeutic Vaccines Institute, Ciudad Bolivar, Venezuela; 2 “Dr. Francisco Battistini Casalta” Health Sciences School, University of Oriente – Bolivar Nucleus, Ciudad Bolivar, Venezuela; 3 Obstetrics and Gynaecology Department, San Cristobal Central Hospital, San Cristobal, Venezuela; 4 “José María Vargas” School of Medicine, Central University of Venezuela, Caracas, Venezuela; 5 “Luis Razetti” School of Medicine, Central University of Venezuela, Caracas, Venezuela; 6 Grupo de Investigación Biomedicina, Faculty of Medicine, Fundación Universitaria Autónoma de Las Américas - Institución Universitaria Visión de Las Américas, Pereira, Risaralda Colombia; 7 Gilbert and Rose-Marie Chagoury School of Medicine, Lebanese American University, Beirut, Lebanon; 8 Master of Clinical Epidemiology and Biostatistics, Universidad Científica del Sur, Lima, Peru; 9 Infectious Diseases Department, University Hospital of Caracas, Caracas, Venezuela

**Keywords:** COVID-19, Pregnancy, Epidemiology, Maternal-foetal outcome, Venezuela

## Abstract

**Background:**

In low- and middle-income countries, pregnant women and newborns are more vulnerable to adverse outcomes from coronavirus disease 2019 (COVID-19). However, in Venezuela, there are no integrated data in a national surveillance system to identify the clinical-epidemiological characteristics and maternal-foetal outcomes of pregnant women hospitalised with COVID-19.

**Methods:**

A retrospective study was conducted among Venezuelan pregnant women hospitalised with COVID-19 seen at the “Ruiz y Páez” University Hospital Complex and the San Cristobal Central Hospital between June 2020 and September 2021. Information was obtained from physical and digitised clinical records using a purpose-designed proforma to collect epidemiological, clinical, paraclinical, treatment, obstetric and perinatal complications, and maternal-foetal outcomes data.

**Results:**

A total of 80 pregnant women with confirmed severe acute respiratory syndrome coronavirus 2 infection were seen within the study period, 59 (73.8%) survived and 21 (26.2%) died. The median (interquartile range) age was 29 (23–33) years, the majority being in the third trimester of pregnancy (81.2%; *n* = 65). Interestingly, four (5%) pregnant women were co-infected with malaria by *Plasmodium vivax* and three (3.8%) with syphilis. The most frequent symptoms were fever (75%; *n* = 60), dry cough (68.8%; *n* = 55), dyspnoea (55%; *n* = 44), and headache (53.8%; *n* = 43). The most frequent maternal complications were anaemia (51.5%; *n* = 66) and hypertensive disorders of pregnancy (17.5%; *n* = 14). The most frequent perinatal complications were preterm delivery (39.2%; *n* = 20/51) and oligohydramnios (31.3%; *n* = 25). A total of 29 (36.3%) adverse foetal outcomes were documented, 21 stillbirth and eight abortions.

**Conclusion:**

This is the first study to describe the clinical-epidemiological behaviour of COVID-19 in hospitalised Venezuelan pregnant women. Anaemia, hypertensive disorders of pregnancy, oligohydramnios, and low birth weight were the most frequent maternal-foetal complications in this population of pregnant women.

## Background

Latin America is one of the most affected regions by the coronavirus disease 2019 (COVID-19) pandemic, showing the highest morbidity and mortality rates in countries with weakened healthcare services, economic and political instabilities, humanitarian crises, and deep social inequalities, such as Venezuela [1–3]. As of 30 November 2022, 547,465 cases and 5,828 deaths from COVID-19 had been reported in Venezuela, some of the lowest reported numbers in the region. However, these data are inconsistent with the real epidemiological behaviour of the pandemic in the country, where underreporting is estimated to be five to seven times higher [4].

Anatomical, physiological, and immunological changes accompanying pregnancy may increase the susceptibility of pregnant women to viral pathogens and the risk of developing severe pneumonia [5]. In addition, studies of other coronavirus infections, such as severe acute respiratory syndrome coronavirus or Middle East respiratory syndrome coronavirus, suggest that infected pregnant women may be more susceptible to adverse outcomes, such as intubation, intensive care unit admission, renal failure, and death [6], as well as an increased risk of neonatal mortality, preterm delivery, and foetal growth restriction [7–9].

Although severe acute respiratory syndrome coronavirus 2 (SARS-CoV-2) is increasingly well understood, outcomes associated with infection during pregnancy remain limited and unclear. At the pandemic’s beginning, scientific reports suggested that pregnant women were not at increased risk of maternal-foetal complications due to COVID-19 [10, 11]. Current knowledge on the impact of SARS-CoV-2 infection in pregnant women has been mainly derived from case reports, case series, and population-based surveillance systems in high-income countries. These data have focused primarily on maternal outcomes in women with symptomatic disease, with maternal death, intrauterine foetal death, and neonatal death reported in approximately 1% of cases in this setting [12–14]. In addition, several studies have described that exposure to COVID-19 increases the likelihood of developing pre-eclampsia/eclampsia, preterm delivery, premature rupture of membrane, and admission to the neonatal intensive care unit [15, 16].

In low- and middle-income countries (LMICs), pregnant women and newborns are more vulnerable to adverse outcomes from COVID-19 [17, 18]. However, to date, there is limited information on the epidemiological and clinical characteristics of COVID-19 in pregnant women in Latin America [19]. In the case of Venezuela, no data is integrated into a national surveillance system to identify maternal-foetal characteristics or the outcomes of hospital admissions of pregnant women for COVID-19. Therefore, this study aimed to evaluate the clinical-epidemiological characteristics and maternal-foetal outcomes in pregnant women with COVID-19 hospitalised in two sentinel centres in Venezuela.

## Methods

### Patients and study design

A retrospective study was conducted including all pregnant women with SARS-CoV-2 infection confirmed by reverse transcriptase polymerase chain reaction (RT-PCR) who were hospitalised in two sentinel centres for the care of patients with COVID-19 in Venezuela, the “Ruiz y Páez” University Hospital Complex, Bolivar state and the San Cristobal Central Hospital, Tachira state, between June 2020 and September 2021. Pregnant women with missing epidemiological, clinical, treatment, obstetric and perinatal complications, and maternal-foetal outcomes data in their medical records, were excluded.

### Data collection

Information was obtained from physical and digitised clinical records using a purpose-designed proforma to collect epidemiological (age, race, provenance by state), clinical (admission symptoms, gynaecobstetric characteristics, pathological history, psychobiological habits, physical examination), paraclinical (haematology, blood chemistry, coagulation tests), treatment (antivirals, antibiotics, antiparasitic, thromboprophylaxis, corticosteroids, oxygen therapy), obstetric (maternal anaemia, hypertensive disorders of pregnancy, pre- and postpartum haemorrhage, maternal sepsis) and perinatal (premature rupture of membranes, placenta praevia, premature detachment of the normally inserted placenta, foetal growth restriction, oligohydramnios) complications, and maternal-foetal outcomes data.

### Statistical analysis

The following descriptive statistics summarised pregnant women’s data: mean, standard deviation (SD), median, interquartile range (IQR), and frequency (%). Kolmogorov–Smirnov test assessed the distribution of variables. Median test was used for variables with a non-normal distribution, and Student’s t-test for those with normal distribution. Pearson’s chi-squared and Fisher’s exact tests were used for categorical variables. When the need for *post-hoc* analysis was detected, the Bonferroni correction of the *p*-value was used to adjust it. *P*-values < 0.05 were considered significant. Due to the small sample size, binomial regression data modelling did not detect risk factors associated with maternal and foetal mortality. Statistical analysis was performed using Statistical Package for the Social Sciences version 26 (International Business Machines Corporation, Armonk, NY, United States). Figures were generated using Microsoft® Excel® version 2019 (Microsoft, Redmond, WA, United States).

## Results

### Epidemiological and gynaecobstetric characteristics and medical history of pregnant women with COVID-19

Eighty pregnant women with SARS-CoV-2 infection confirmed by RT-PCR were included. Of these, 59 (73.8%) survived and 21 (26.2%) died. The median (IQR) age of pregnant women was 29 (23–33) years; most were of mixed race (85%; *n* = 68) and in the third trimester of pregnancy (81.2%; *n* = 65). No statistically significant differences were found between the epidemiological and gynaecobstetric characteristics of the surviving and dead pregnant women (Table 1). The most frequent co-morbidity was obesity (27.5%; *n* = 22), followed by hypertension (3.8%; *n* = 3) and diabetes (3.8%; *n* = 3). Interestingly, four (5%) pregnant women were co-infected with malaria by *Plasmodium vivax* and three (3.8%) with syphilis. Obesity was more common in dead pregnant women (38.1%) compared to survivors (23.7%); however, no statistically significant differences were found (*p* = 0.205).

**Table 1:** Epidemiological and gynaecobstetric characteristics and medical history of 80 pregnant women with confirmed SARS-CoV-2 infection in Venezuela

	All (***N*** = 80)	Survivors (***N*** = 59)	Dead (***N*** = 21)	***p***-value
*Epidemiological characteristics*				
Age, median (IQR), years	29 (23–33)	28 (23–33)	29 (23–33)	0.464^*^
Age group, *n* (%)				0.946^†^
≤ 20 years	15 (18.8)	11 (18.6)	4 (19)	
21–30 years	36 (45)	26 (44.1)	40 (47.7)	
≥ 31 years	29 (36.2)	22 (37.3)	7 (33.3)	
Race, *n* (%)				0.507^†^
Mixed	68 (85)	50 (84.7)	18 (85.7)	
White	6 (7.5)	4 (6.8)	2 (9.5)	
Black	4 (5)	4 (6.8)	0 (0)	
Indigenous	2 (2.5)	1 (1.7)	1 (4.8)	
Provenance by state, *n* (%)				0.138^‡^
Bolivar	47 (58.8)	31 (52.5)	16 (76.2)	
Tachira	30 (37.5)	25 (42.4)	5 (23.8)	
Anzoategui	3 (3.7)	3 (5.1)	0 (0)	
*Gynaecobstetric characteristics*				
Trimester of pregnancy, *n* (%)				0.305^‡^
First trimester	3 (3.8)	2 (3.4)	1 (4.8)	
Second trimester	12 (15)	11 (18.6)	1 (4.8)	
Third trimester	65 (81.2)	46 (78)	19 (90.4)	
No. of previous pregnancies, *n* (%)				0.732^†^
0	20 (25)	16 (27.1)	4 (19)	
1–3	54 (67.5)	39 (66.1)	15 (71.4)	
4–6	6 (7.5)	4 (6.8)	2 (9.5)	

^*^Median test

^†^Pearson’s chi-square test

^‡^Fisher’s exact test

*IQR* interquartile range

### Clinical characteristics of pregnant women with COVID-19

**Fig. 1:**
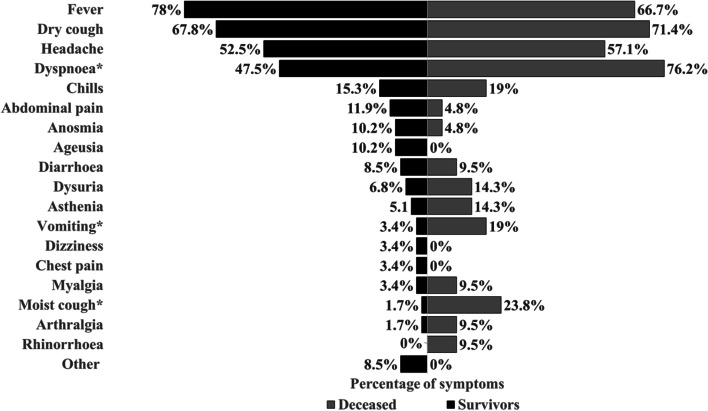
Symptoms of 80 pregnant women with confirmed SARS-CoV-2 infection in Venezuela. *p < 0.05 (*p*-values by Pearson’s chi-square).

The median (IQR) time from symptom onset to hospitalisation was 6 (2–8) days, with no statistically significant difference between surviving and dead pregnant women (*p* = 0.47). The median (IQR) time from symptom onset to labour was 12 (7–18.5) days. The median (IQR) time from symptom onset to maternal death was 7 (6–16) days. Fever (75%; *n* = 60), dry cough (68.8%; *n* = 55), dyspnoea (55%; *n* = 44), and headache (53.8%; *n* = 43) were the most frequent symptoms in all pregnant women; the least frequent symptoms were dizziness (2.5%; *n* = 2), chest pain (2.5%; *n* = 2), and rhinorrhoea (2.5%; *n* = 2). A higher proportion of dyspnoea, vomiting, and wet cough were found in dead pregnant women compared to survivors (76.2% vs. 47.5%, *p* = 0.023; 19% vs. 3.4%, *p* = 0.038; 23.8% vs. 1.7%, *p* = 0.004; respectively) (Fig. 1).

On physical examination, median heart rate and median respiratory rate were significantly higher in dead pregnant women compared with survivors (101 [86–112] vs. 85 [80–98], *p* = 0.015; 24 [20–29] vs. 19 [18–22], *p* = 0.007; respectively), while median oxygen saturation was significantly higher in surviving pregnant women compared to dead (94 [90–97] vs. 81 [72–89], *p* < 0.001). A higher proportion of oxygen saturation ≥ 94% was found in surviving pregnant women compared to dead (54.2% vs. 14.3%, *p* < 0.001), while a higher proportion of oxygen saturation < 80% was found in dead pregnant women compared to survivors (47.6% vs. 6.8%, *p* < 0.001). Altered consciousness was more frequent in dead pregnant women than in those who survived (5% [*n* = 4] vs. 1.25% [*n* = 1], *p* = 0.016).

### Paraclinical findings of pregnant women with COVID-19

Table 2 shows the paraclinical findings. No statistically significant differences were observed between the paraclinical results of surviving and dead pregnant women. Median lactate dehydrogenase was higher in dead pregnant women than in survivors (482 [297–1520] vs. 258 [163–557] mg/dL); however, there was no statistically significant difference (*p* = 0.263).

**Table 2:** Paraclinical findings in 80 pregnant women with confirmed SARS-CoV-2 infection in Venezuela

	All (***N*** = 80)	Survivors (***N*** = 59)	Dead (***N*** = 21)	***p***-value
Haemoglobin, median (IQR), g/dL (*n* = 66)	10.9 (9.8–11.5)	11 (9.8–11.8)	10.7 (9.7–11)	0.619^*^
Haematocrit, median (IQR), % (*n* = 66)	33 (29.6–35.9)	33 (30–36)	32 (29.4–34)	0.694^*^
Leukocytes, median (IQR), × 10^3^/mL (*n* = 66)	9.5 (7.7–12.9)	9.5 (7.7–12.9)	9.2 (8.3–12.6)	1^*^
Neutrophils, mean (SD), ×10^3^/mL (*n* = 66)	78 (10)	78 (10)	82 (8)	0.61^†^
Lymphocytes, mean (SD), ×10^3^/mL (*n* = 66)	21 (10)	20 (11)	17 (16)	0.63^†^
Platelets, median (IQR), ×10^3^/mL (*n* = 66)	185 (155–222)	187 (165–220)	150 (138–245)	0.619^*^
Glycaemia, mean (SD), mg/dL (*n* = 46)	93 (19)	89 (18)	103 (18)	0.769^†^
Urea, median (IQR), mg/dL (*n* = 36)	20.5 (15.5–34)	20 (14–28.5)	22 (15–45)	0.289^*^
Creatinine, median (IQR), mg/dL (*n* = 36)	0.8 (0.7–1)	0.8 (0.7–0.9)	0.9 (0.7–1.4)	0.724^*^
PT, median (IQR), seconds (*n* = 14)	12.4 (12–15)	12.9 (11.3–15.8)	12.0 (11.3–12.8)	0.559^*^
PTT, median (IQR), seconds (*n* = 14)	31.4 (30–33.2)	32.4 (30–33.2)	29 (27.5–32.5)	0.263^*^
AST, mean (SD), mg/dL (*n* = 13)	51.5 (19.2)	50.4 (20.8)	57.5 (4.9)	0.152^†^
ALT, mean (SD), mg/dL (*n* = 13)	49.3 (23.8)	51.2 (25.4)	38.5 (7.8)	0.194^†^
LDH, median (IQR), mg/dL (*n* = 53)	301 (180–587)	258 (163–557)	482 (297–1520)	0.263^*^
D-dimer, median (IQR), ng/dL (*n* = 42)	19.0 (11.1–22.9)	19.2 (11.1–22.6)	18.9 (12.9–27.2)	0.717^*^
Ferritin, median (IQR), ng/dL (*n* = 42)	288 (180–450)	280 (180–410)	320 (200–689)	0.452^*^

^*^Median test

^†^Student’s t-test

*IQR* interquartile range, *PT* prothrombin time, *PTT* partial thromboplastin time, *LDH* lactate dehydrogenase

### Maternal and perinatal complications and foetal outcomes of pregnant women with COVID-19

The most frequent maternal and perinatal complications were anaemia (51.5%; *n* = 66), oligohydramnios (31.3%; *n* = 25), and hypertensive disorders of pregnancy (17.5%; *n* = 14) (Table 3).

**Table 3:** Maternal and perinatal complications in 80 pregnant women with confirmed SARS-CoV-2 infection in Venezuela

	All (***N*** = 80)	Survivors (***N*** = 59)	Dead (***N*** = 21)	***p***-value
*Maternal complications, n (%)*				
Anaemia	66 (51.5)	53 (49.1)	13 (61.5)	0.420^*^
Hypertensive disorders of pregnancy	14 (17.5)	12 (20.3)	2 (9.5)	0.263^*^
*Perinatal complications, n (%)*				
Oligohydramnios	25 (31.3)	22 (37.3)	3 (14.3)	0.051^*^
Premature rupture of membranes	5 (6.3)	5 (8.5)	0 (0)	0.208^†^
Foetal growth restriction	3 (3.8)	3 (5.1)	0 (0)	0.396^†^
Placenta praevia	1 (1.3)	1 (1.7)	0 (0)	0.737^†^
Premature detachment of the normally inserted placenta	1 (1.3)	1 (1.7)	0 (0)	0.737^†^

^*^Pearson’s chi-square test

^†^Fisher’s exact test

Almost all pregnant women received antibiotic therapy (96.3%; *n* = 77), the most frequent being Ceftriaxone (63.6%; *n* = 49), followed by Vancomycin (59.7%; *n* = 46), and Cephalothin (16.8%; *n* = 13). Pregnant women with *Plasmodium vivax* co-infection received different antiparasitic drugs for malaria treatment (Chloroquine: 3.8%, and Artesunate injection: 1.3%). A higher proportion of anticoagulant use (Enoxaparin) was found in dead pregnant women compared to survivors (81% vs. 55.9%, *p* = 0.042). Regarding corticosteroids, 37.5% (*n* = 30) of pregnant women received Dexamethasone or Methylprednisolone, with no statistically significant difference between surviving and dead pregnant women (*p* = 0.595). Maternal non-invasive mechanical ventilation was necessary for eight pregnant women (10%), and one required invasive mechanical ventilation (1.3%).

There were 29 adverse foetal outcomes, 21 (26.2%) stillbirth and eight (10%) abortions. A higher proportion of abortions was found in dead pregnant women compared to survivors (23.8% vs. 5.1%, *p* = 0.014). Of the live newborns, most were delivered by caesarean section (92.2%; *n* = 47/51), preterm (39.2%; *n* = 20/51) and with low birth weight (53.6%; *n* = 27/51); however, with suitable adaptation status (7–10 points) at the fifth (86.3%; *n* = 44/51) minute by APGAR score. Term delivery was more frequent in survivors compared to the dead (*p* = 0.026). Severely depressed live births at the fifth minute by APGAR score were more frequent in dead pregnant women than in survivors (37.5% [*n* = 3] vs. 0% [*n* = 0], *p* < 0.001) (Table 4). Only eight newborns survived out of the 21 pregnant women died.

**Table 4:** Foetal outcomes in 80 pregnant women with confirmed SARS-CoV-2 infection in Venezuela

	All (***N*** = 80)	Survivors (***N*** = 59)	Dead (***N*** = 21)	***p***-value
*Foetal outcome, n (%)*				
Stillbirth	21 (26.2)	13 (22)	8 (38.1)	0.151^*^
Abortion	8 (10)	3 (5.1)	5 (23.8)	0.014^*^
Live birth	51 (63.8)	43 (72.9)	8 (38.1)	
Type of delivery, *n* (%) (*n* = 51)				0.494^†^
Caesarean section	47 (92.2)	39 (90.7)	8 (100)	
Vaginal	4 (7.8)	4 (9.3)	0 (0)	
Gestational age at delivery, *n* (%) (*n* = 51)				0.026^*^
Term (37–41^+ 6^)	31 (60.8)	28 (65.1)	3 (37.5)	
Late preterm (34–36^+ 6^)	7 (13.7)	6 (14)	1 (12.5)	
Moderate preterm (32–33^+ 6^)	7 (13.7)	4 (9.3)	3 (37.5)	
Very preterm (28–31^+ 6^)	5 (9.8)	5 (11.6)	0 (0)	
Extreme preterm (< 28)	1 (2)	0 (0)	1 (12.5)	
Birth weight, *n* (%) (*n* = 51)				0.352^*^
Normal weight (2500–3750 g)	24 (47.1)	22 (51.2)	2 (25.0)	
Low birth weight (1500–2499 g)	21 (41.8)	16 (37.2)	5 (62.5)	
Very low birth weight (1000–1499 g)	6 (11.8)	5 (11.6)	1 (12.5)	
APGAR score at 5th minute, *n* (%) (*n* = 51)				
7–10	44 (86.3)	40 (93.0)	4 (50.0)	0.001^*‡^
4–6	4 (7.8)	3 (7.0)	1 (12.5)	0.596^*‡^
0–3	3 (5.8)	0 (0.0)	3 (37.5)	< 0.001^*‡^

^*^Pearson’s chi-square test

^†^Fisher’s exact test

^‡^Statistically significant *p*-value according to Bonferroni correction = 0.008

## Discussion

This study describes the primary maternal and foetal outcomes among pregnant women hospitalised with COVID-19 in two sentinel centres in Venezuela. The median age and trimester of pregnancy at the presentation of our pregnant women were similar to those reported in other studies [20, 21]. Obesity, hypertension, and diabetes were observed in similar frequencies to previously documented [21–23]. A group of pregnant women reported co-infection with malaria and syphilis, in accordance with co-infections reported in LMICs [17, 24]. Consistent with previous publications [20, 21, 25, 26], our study found that fever, headache, cough, and dyspnoea were the most frequent symptoms reported by pregnant women with COVID-19 at consultation and hospital admission.

Overall estimates in low-risk pregnancies for pre-eclampsia and eclampsia are 4.6 and 1.4%, respectively, with wide variation between regions. In this study, hypertensive pregnancy syndrome was the most frequent maternal complication (17.5%), similar to previous reports [21, 27]. Hypertensive disorders of pregnancy and COVID-19 share an exaggerated inflammatory response, guided by cytokines such as IL-6, TNF-α and IFN-γ leading to endothelial damage [28]. Likewise, studies in Latin America [21] and other regions of the world [14–16, 29] found that preterm delivery and low birth weight were the most frequent perinatal complications in our patient population, which could be associated with excessive host inflammatory response to COVID-19 and poor foetal vascular perfusion of the placenta [14]. In our study, oligohydramnios was documented in almost one-third (31.3%) of pregnant women, which contrasts with other studies [21], where an incidence of 3% (10 times lower) was reported. That could be explained by the measurement variability of this parameter, which depends on the operator/echographer performing it in each institution. Nevertheless, this study demonstrated an increased risk of maternal and perinatal morbidity and mortality associated with SARS-COV-2 infection, likely due to difficult access to health care and reduced healthcare workers and intensive care unit beds in LMICs, such as Venezuela.

Although the low frequency of bacterial superinfection in patients with COVID-19 (6.9–8%) is documented [30, 31], our study reported that almost all (96.3%) pregnant women received at least one course of antibiotic therapy during their hospitalisation. However, indiscriminate antibiotic use during the pandemic has been the common denominator (71–90%) [31], a clinical practice that increases bacterial multidrug resistance [30, 32, 33]. On the other hand, the prothrombotic risk in pregnant women with SARS-CoV-2 infection has been reported previously [34]. Therefore, particular recommendations have been made on using antithrombotic prophylaxis or anticoagulation in pregnant women [35]. We found here a higher proportion of anticoagulant use in dead pregnant women than in survivors. However, it is impossible to conclude because it is necessary to consider unassessed variables such as dose, disease severity, and even the timing of the pandemic, as the role and indication of antithrombotics in managing COVID-19 were unknown at the beginning of the pandemic.

Among the maternal outcomes, we found an extremely high case fatality ratio (26.2%) compared with similar studies [16, 36], associated with the presence of poor prognosis markers such as elevated lactate dehydrogenase, C-reactive protein, ferritin, and D dimer, as well as thrombocytopenia and lymphopenia [30, 37–49]. An increased risk of abortions and stillbirth in patients with COVID-19 has been described in larger studies [17, 50, 51], a pattern observed in our study with a higher proportion within the dead pregnant women, similar to that described in a Latin-American study [21], with the exception of the proportion of stillbirth. A possible hypothesis that could explain these discrepancies is poor antenatal care due to the collapse of health care centres during the COVID-19 pandemic in Venezuela, as well as quarantine and community containment measures [52]. Additionally, the retrospective nature of our study, which only included hospitalised pregnant women, and the limited access to routine screening for SARS-CoV-2 infection among pregnant women, may have contributed to this percentage difference in mortality.

There is evidence that corticosteroids reduce mortality, hospitalisation time, and use of mechanical ventilation in COVID-19 patients with supplemental oxygen requirements [53], so they have been applied as an indispensable tool, even in pregnant women [54]. In this study, more than one-third of pregnant women (37.5%) received some form of corticosteroid; however, the number is lower than that of pregnant women with supplemental oxygen requirements (52.5%). There is substantial evidence to support the use of corticosteroids in pregnant women at risk of preterm delivery [30], one of the complications with the highest incidence in our study.

This study has multiple limitations. In the first place, the design of the retrospective study did not allow obtaining the direct cause of maternal death, maternal COVID-19 vaccination status, total number of maternal admissions in the study period or socio-economic data due to the deterioration of the statistical recording system and medical records in the two sentinel sites of our study. Similarly, not all the paraclinical studies of pregnant women were obtained, limiting their analysis. Second, it includes a small number of patients, which limits the multivariable analysis design for identifying risk factors associated with mortality. Third, despite including pregnant women from two sentinel centres for the care of patients with COVID-19, our results cannot be extrapolated to the general population since the availability of intensive care beds varies between different hospitals as the complexity of caring for pregnant women. Finally, it is possible that the clinician’s decision was decisive at the time of patient admission, leading him to admit pregnant women with COVID-19 with mild symptoms only for surveillance and foetal evaluation, so the additional admission criteria of each institution and even between each specialist limit the interpretation of our results. Therefore, prospective, longitudinal, and multicentre studies are needed to understand better the factors associated with maternal mortality from COVID-19 in Venezuela.

## Conclusions

This study describes the clinical-epidemiological behaviour of COVID-19 in hospitalised Venezuelan pregnant women. Anaemia, hypertensive syndrome of pregnancy, oligohydramnios, and low birth weight were the most frequent maternal-foetal complications in our patient population. Therefore, it is recommended to establish a national surveillance system for pregnant women with COVID-19 and to carry out multicentre studies to identify risk factors associated with mortality in this patient population.

## Data Availability

All data generated or analysed during this study are included in this article.

## Abbreviations

COVID-19: Coronavirus disease 2019
SARS-CoV-2: Severe acute respiratory syndrome coronavirus 2
RT-PCR: Reverse transcriptase polymerase chain reaction
SD: Standard deviation
IQR: Interquartile range
